# An elderly patient presenting with a primary spinal multifocal intradural extramedullary pilocytic astrocytoma: a case report and review of the literature

**DOI:** 10.1186/s12885-018-4721-y

**Published:** 2018-08-09

**Authors:** Devin McBride, Zaid Aljuboori, Eyas M. Hattab, Richard Downs, Shiao Woo, Brian Williams, Joseph Neimat, Eric Burton

**Affiliations:** 1 University of Louisville School of Medicine, Louisville, KY 40202 USA; 2 Departments of Pathology, University of Louisville School of Medicine, Louisville, KY 40202 USA; 3 Departments of Neurosurgery, University of Louisville School of Medicine, Louisville, KY 40202 USA; 4 Departments of Radiation Oncology, University of Louisville School of Medicine, Louisville, KY 40202 USA; 5 Departments of Radiology, University of Louisville School of Medicine, Louisville, KY 40202 USA; 6 Departments of Neurology, University of Louisville School of Medicine, Louisville, KY 40202 USA

**Keywords:** Pilocytic astrocytoma, Adult, Extramedullary glioma

## Abstract

**Background:**

Pilocytic astrocytoma is a low-grade central nervous system tumor most commonly seen in children. Dissemination from a primary intracranial tumor along the neuroaxis has been described at both presentation and disease progression. However, the development of an intradural extramedullary pilocytic astrocytoma independent of a primary intraparenchymal tumor in an adult patient with no history of pilocytic astrocytoma has rarely been reported.

**Case presentation:**

A 69-year-old woman presented with progressive myelopathic symptoms and thoracic radicular pain. MRI imaging of the whole spine showed an enhancing intradural extramedullary lesion extending from the cervical cord to T11 causing cord compression. Laminectomies were performed for surgical decompression and histopathology was consistent with pilocytic astrocytoma. Complete staging was done that included imaging of the brain and cerebrospinal fluid cytology. No other tumor was found by these methods. Postoperatively the patient was treated with large field spinal radiation and concurrent chemotherapy followed by adjuvant chemotherapy. She has thus far been clinically and radiographically stable.

**Conclusion:**

This is a rare case of an adult with multiple spinal pilocytic astrocytomas in an intradural extramedullary location, typically the result of cerebrospinal fluid dissemination of neoplastic cells from a primary intracranial tumor site (i.e. drop metastasis). No conventional primary tumor was identified in this patient, suggesting these tumors may arise from heterotopic gliomas.

## Background

Pilocytic astrocytoma (PA) is the most common central nervous system (CNS) tumor affecting the pediatric population but is rare in older adults [1]. It corresponds histologically to a WHO grade I tumor and is generally well circumscribed and slow growing [2]. The tumor most commonly arises in the cerebellum, brain stem, hypothalamus, optic nerve or in the intramedullary spinal cord, and complete surgical resection when possible can be considered curative [3, 4]. Craniospinal axis dissemination of pilocytic astrocytoma is a rare event that likely occurs via cerebrospinal fluid (CSF) pathways as a primary mechanism [5–7]. When this happens, distal tumor can present in the intradural extramedullary space as either discrete nodular disease, i.e., drop metastasis or as diffuse leptomeningeal gliomatosis [8–11].

Here we present a highly unusual multifocal intradural extramedullary pilocytic astrocytoma of the spine in an older adult. The location of the tumor in this patient suggested they were drop metastasis from a primary tumor, however imaging of the entire craniospinal axis revealed no other tumor site. This is a rarely reported presentation of pilocytic astrocytoma. In this report we discuss the case and review the relevant literature with a focus on possible mechanisms.

## Case presentation

The patient is a 69-year-old woman who presented to her primary care physician with worsening cough and pain wrapping around her chest to her back. The chest pain was initially thought to be pleuritic in nature and related to an exacerbation of her chronic obstructive pulmonary disease (COPD). When there was no improvement in her symptoms with treatment of her COPD, and considering the confounding radicular distribution of her pain, a plan was made to have an MRI of the spine performed to explore other possible etiologies of her pain.

Prior to the scheduled MRI, the patient was seen in the emergency department and diagnosed with a myocardial infarction due to hypertensive emergency. During her admission to the hospital, the patient was noted to be experiencing neck pain, bilateral upper extremity pain in a C8-T1 dermatomal distribution and a loss of urinary and fecal urges. On examination, the patient’s force of flexion and abduction in her upper extremities were mildly reduced. The strength in her lower extremities was decreased. Her deep tendon reflexes were 3+ in her bilateral upper extremities and 3+ in her bilateral lower extremities with clonus at the ankles.

She had bilateral extensor plantar responses. In light of these progressive myelopathic symptoms a spinal MRI was done.

**Fig. 1:**
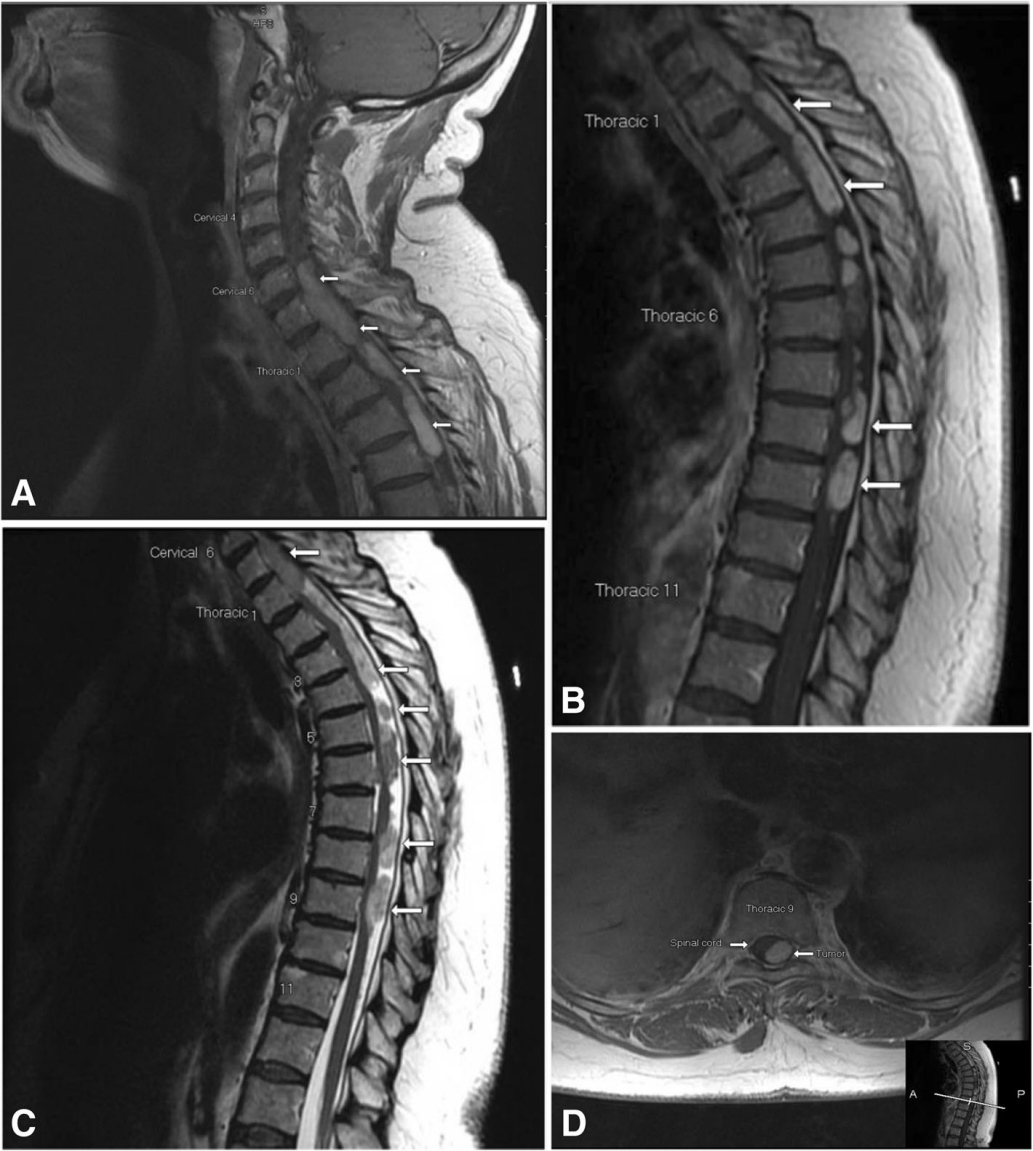
Magnetic resonance imaging of cervical - thoracic intradural extramedullary pilocytic astrocytoma. Initial MRI imaging of the cervical and thoracic spine, **a** Sagittal post contrast T1 cervical spine, **b.** Sagittal post contrast T1 thoracic spine**, c.** Sagittal T2 thoracic spine, demonstrates multiple enhancing intradural extramedullary tumors in the lower cervical and upper and middle thoracic spinal canal (white arrows indicate tumor). These cause multifocal severe spinal canal stenosis with marked flattening of the spinal cord, **d.** Axial post contrast T1 at level of T9

The initial pre and post -contrast MRI of the entire spine showed extensive, mostly brightly enhancing intradural extramedullary nodules extending from C5–6 to T11 (Fig. 1). Some nodules at the T6 level were only minimally enhancing, with pre-contrast T1 hyperintensity apparent. The lesions were causing multilevel severe central canal stenosis with multilevel cervical and thoracic spinal cord compressions. The largest mass conglomeration extended from C6 to T4, measuring up to 1.2 cm AP × 1.9 cm transverse × 10.0 cm CC. Despite the extensive mass effect with flattening of the spinal cord, the spinal cord demonstrated no intramedullary T2 hyperintensity except for a small focus of enhancing T2 hyperintensity at the posterior columns at the T7-T8 level that could have represented a small area of invasive change or perhaps focal indentation by a focal nodular component of the large extramedullary mass.

The multifocal spinal masses were suspicious for leptomeningeal carcinomatosis and imaging was ordered to look for a primary tumor source. An MRI of the brain showed no tumor although there was a small nonspecific non-enhancing extra-axial nodule in the left side prepontine cistern at the level of the upper pons. The patient’s computed tomography (CT) of the chest, abdomen and pelvis showed no tumor, and two CSF specimens were also negative for neoplastic cells. A follow-up MRI of the spine was performed ten days later to determine the patient’s response to steroid therapy and this showed no significant change. Multiple spinal meningiomas were also considered due to the patient’s age, the intradural extramedullary location and the homogeneous enhancement seen on MRI.

Of note, the patient did not have a history of neurofibromatosis or any other neurocutaneous disorders. The patient also did not have a past medical history of tumors and was never previously treated with chemotherapy or radiation. The patient does have a history of polio which she recalled contracting at about two years of age. The patient’s mother was diagnosed with a fast-growing brain tumor in her 80s but otherwise the patient’s family history was unremarkable.

### Surgical intervention

**Fig. 2:**
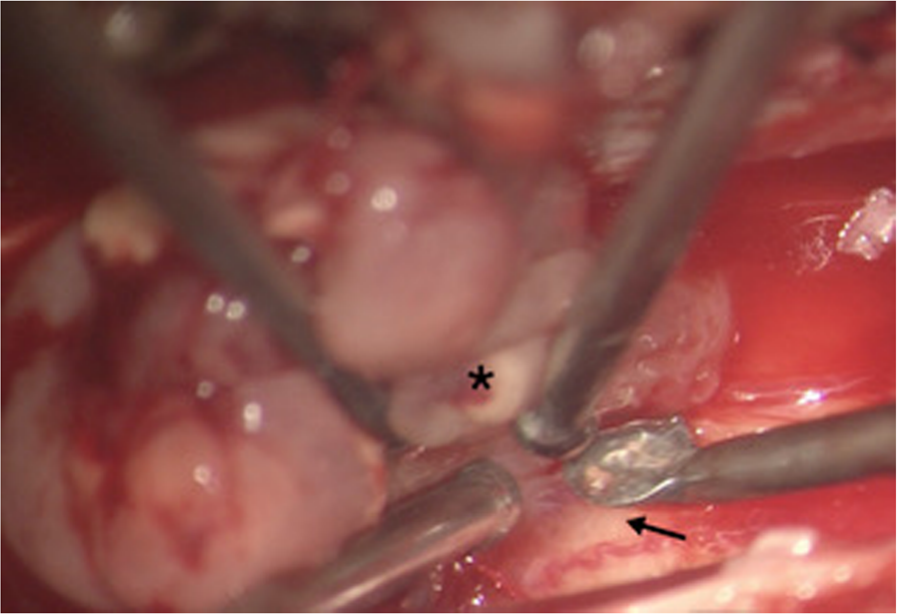
Intraoperative image**.** Intraoperative image (magnification 5×) showing tumor (*) with an identifiable plane, easily separated from normal spinal cord **(↑**)

An initial T7-T8 laminectomy for partial excision of the tumor was done for tissue diagnosis. Once the lesion was determined to be a pilocytic astrocytoma, a decision was made to proceed with an extensive resection of the tumor. The residual tumor was removed in two stages. In the first surgery a multilevel laminectomy was done from T4-T10. After a midline dural opening was made, tumor was identified which was light silver in color and attached to the spinal cord (Fig. 2). A careful microsurgical dissection was done to separate the tumor from the spinal cord. There was an identifiable plane between tumor and the cord which facilitated safe removal of the tumor, with the exception of certain areas where the tumor seemed to be more adherent and possibly invaded the pial covering of the spinal cord. At these locations the tumor was shaved off the spinal cord leaving only a very thin layer of tumor. Later a C4-T3 laminectomy was done for the second resection and removal of the remaining tumor.

### Pathology

**Fig. 3:**
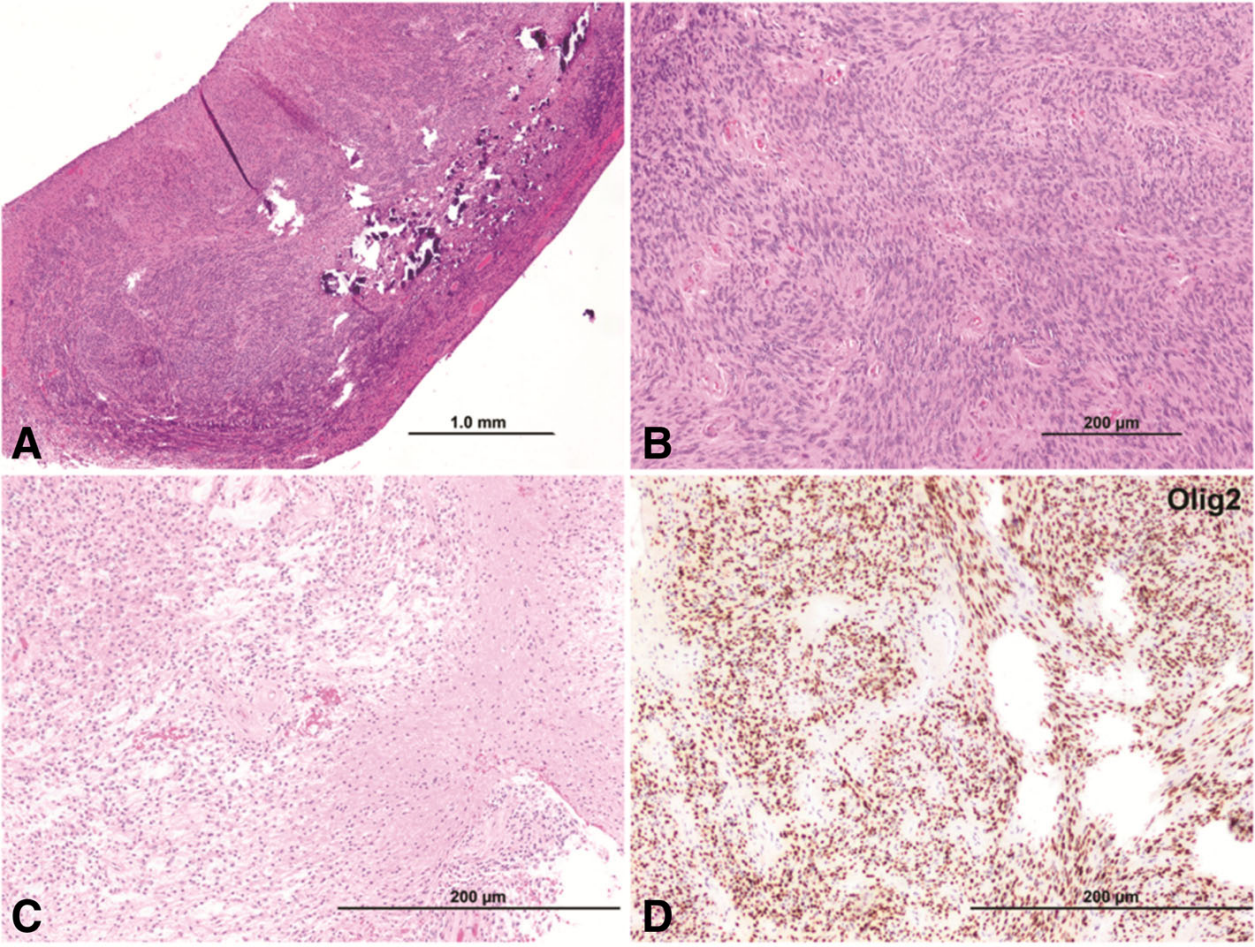
Histopathology of adult pilocytic astrocytoma**.** Pilocytic astrocytoma with anaplastic features. The tumor consists of multiple well-circumscribed nodules, some with dense calcification. **a** Some nodules consist of tightly packed spindle cells with hyperchromatic nuclei (magnification 2× hematoxylin and eosin [H&E]). **b** Mitoses are readily identified in these areas (magnification 10× H&E). Note the perivascular pseudorosettes. Other nodules exhibit conventional pilocytic astrocytoma morphology with (**c**) the classic biphasic pattern (magnification 20× H&E). The tumor cells are strongly immunoreactive for S100, GFAP, and (**d**) Olig2 (magnification 20× H&E)

Routine hematoxylin and eosin–stained sections of the tumor samples showed multiple well-circumscribed masses made up predominantly of interlacing fascicles of spindle cells with thin wavy nuclei. The degree of cellularity varied greatly with some nodules exhibiting highly cellular compact areas and others showing more loosely arranged cells. Perivascular pseudorosettes were prominent in some areas, giving an ependymoma-like appearance. Foci of dense calcifications and microcystic degeneration were identified. Mitoses were elevated (up to 8 per 10 high power fields in the most cellular areas), nuclei were hyperchromatic, and the proliferation index was high in those areas signifying the presence of anaplastic features. The tumor cells were strongly and diffusely immunoreactive for S100, GFAP, and Olig2, confirming their glial nature. They were negative for EMA and progesterone receptors (meningioma markers) as well as CK18 (usually positive in ependymomas). SOX10, a marker of peripheral nerve sheath tumors, was also negative. The diagnosis of pilocytic astrocytoma with focal anaplastic features was rendered (Fig. 3).

### Postoperative course and treatment

The patient presented for a physician visit a month after the final surgical procedure. She was experiencing paresthesias in all four extremities, in addition to thoracic radicular type numbness bilaterally at T5. The patient was participating in physical therapy and had persistent bilateral lower extremity weakness.

Radiotherapy with concurrent temozolomide chemotherapy was given to treat the residual tumor. The patient was treated with large field radiation to the spinal cord, receiving a total dose of 36 Gy in 20 fractions with daily dose concurrent temozolomide. Maintenance temozolomide was started, with a plan to treat the patient for six additional cycles. At the time of this writing it has been 13 months since her symptoms first began and six months since her final surgery.

## Discussion and conclusions

We present a case of an adult patient with a spinal intradural extramedullary multifocal pilocytic astrocytoma. The radiographic differential diagnosis for primary CNS tumors in this anatomic location is commonly meningioma or nerve sheath tumors [12]. In this patient the tumor was thought to represent spinal drop metastasis from a primary intracerebral PA. However no other tumor was found in the conventional primary tumor locations when the entire craniospinal axis was imaged by MRI. The patient was treated after resection with large field radiation and concurrent temozolomide chemotherapy and is now completing maintenance temozolomide.

In 1951 Cooper et al. published one of the first series of primary extramedullary gliomas [13]. They describe 15 cases of “extramedullary gliomas along the spinal axis, but without any apparent attachment thereto, and which have not arisen by seeding from a primary intramedullary glial or intracerebral neoplasm”. The average age of their patients was 27 years and of the 15 cases, 9 were ependymoma, 5 were grade I astrocytoma and the remaining case was a grade II astrocytoma. The authors concluded that these were “heterotopic” gliomas developed from subarachnoid glial heterotopias that occurred during development [14].

However, given that the Cooper series is of patients treated between 1915 and 1941, the presence of an asymptomatic undiagnosed primary tumor in these cases is uncertain, although the authors do state that, “cases with any doubt as to their primary extramedullary origin were eliminated from the study”. Their report then is one of the first to suggest, that primary intradural extramedullary glial tumors can occur and should be considered as a rare possibility. This finding is supported by two, more modern case series describing primary intradural extramedullary gliomas published by Venkataramana et al. and Dinakar et al. which included MR imaging [15, 16].

Specific to spinal extramedullary pilocytic astrocytoma in adults, in 2005 Bohner et al. reported on a case of a 25 year old male with no previous diagnosis of PA, who presented with primary diffuse leptomeningeal gliomatosis with confirmed pilocytic astrocytoma histology [17]. This patient had a 3 month history of ascending paresthesias and MRI imaging of the spine revealed an intradural extramedullary nodular mass which affected the whole spine. The initial tissue diagnosis was made by biopsy and the patient was treated with chemotherapy. Unfortunately this patent died 5 months after tumor presentation from his disease. But unique in this instance an autopsy was performed to look for a primary neoplasm in the brain, spine or optic nerve, and none was found. In this case a definitive diagnosis of primary leptomeningeal PA could be made since tumor was found only in the subarachnoid space and no part of the glioma was detected in central nervous system tissue by autopsy. These authors, like Cooper et al., also concluded that pilocytic astrocytoma could arise from heterotopic glial cells in the leptomeninges. Similar to our older patient, Basheer et al. published a case in 2017 of a 56-year-old woman with a multifocal intradural extramedullary pilocytic astrocytoma of the spinal cord with no other primary tumor found. This patient was treated with resection alone and was doing well at 8 months follow-up [18].

It is appropriate to consider and evaluate patients with pilocytic astrocytoma for known tumor predisposition syndromes like neurofibromatosis type 1 (NF1). NF1 is associated with an increased risk of CNS malignancies, and common among CNS tumors in NF1 patients are low grade gliomas, with PA being one of the most common subtypes [19]. Although this patient had no formal genetic testing to exclude NF1, she met none of the clinical criteria for the disease. Interestingly none of the patients cited in this report were noted to have NF1 or any other tumor predisposition syndrome, suggesting these tumors may have oncogenic molecular mechanisms more closely related to pilocytic astrocytoma that develop sporadically [20].

Definitive treatment recommendations cannot be made based on this small sample size. In our case of a nodular spinal tumor with cord compression, the decision was made to opt for maximal safe resection followed by combined chemoradiation given the patients age and the focal anaplastic histologic features of the tumor.

This is an unusual case of an older adult with a pilocytic astrocytoma found in the intradural extramedullary space. This finding supports previous research that suggests gliomas originating in this anatomic location may result from glial cell heterotopias. Genetic studies could shed light on how these tumors develop as well as their clinical course.

## Abbreviations

CNS: Central nervous system
COPD: Chronic obstructive pulmonary disease
CSF: Cerebrospinal fluid
MRI: Magnetic resonance imaging
NF1: Neurofibromatosis type 1
PA: Pilocytic astrocytoma
